# First‐Trimester Down Syndrome Screening in Renal‐Transplanted Pregnant Women: Blood Creatinine Levels Impact False‐Positive Rate

**DOI:** 10.1002/pd.70124

**Published:** 2026-03-19

**Authors:** Clément Burgy, Lisa Chanourdie, Béatrice Guyard‐Boileau, Anne‐Laure Hebral, Annelise Genoux, Marie‐Sophie Combis, Paul Guerby, Christophe Vayssiere, Safouane M. Hamdi

**Affiliations:** ^1^ Laboratoire de Biochimie Institut Fédératif de Biologie CHU de Toulouse Toulouse France; ^2^ Service d’Obstétrique Pôle Femme‐Mère‐Couple CHU de Toulouse Toulouse France; ^3^ Service de Néphrologie et Transplantation d'organes CHU de Toulouse Toulouse France; ^4^ Univ Toulouse, INSERM, CERPOP, BIOETHICS Université Paul‐Sabatier Toulouse France

## Abstract

**Objective:**

Over the past few decades, several thousand pregnancies following kidney transplantation have been monitored but few studies have reported on first‐trimester Down syndrome screening. This study aimed to assess the performance of such screening and its relationship with renal function.

**Method:**

We retrieved data on 16 pregnancies after kidney transplantation and 30 controls matched for age, weight and gestational age. First‐trimester blood creatinine levels were systematically researched. Among other variables, multiples of the median (MoM) values for free‐βhCG, pregnancy‐associated plasma protein‐A (PAPP‐A), nuchal translucency were compared. After statistical review, we combined our cases group with that of a previous study to obtain a cohort of 27 post‐transplantation pregnancies to explore correlations between variables.

**Results:**

Median values of fβ‐hCG MoM of cases and controls were significantly different (2.02 vs. 0.92; *p* < 0.001) whereas those of PAPP‐A and NT MoM were not. Screen positive rate (SPR) were respectively 44% and 17% (cut‐off 1/1000). Linear regressions were observed for fβ‐hCG MoM and creatinine, but not for PAPP‐A, in our initial case group (*r* = 0.699, *p* = 0.025) and the merged group (*r* = 0.724, *p* < 0.001).

**Conclusion:**

We confirmed a specific profile for first‐trimester screening variables in first‐trimester post‐transplantation pregnancies and the impact of renal function, through creatinine levels, on SPR.

## Summary

- What is already known about this topic?
  ◦ The false positive rate (FPR) is high for Down syndrome first‐trimester screening in women who have received kidney transplants.
- What does this study add?
  ◦ This high FPR is due to a specific profile: high free‐βhCG but normal PAPP‐A and nuchal translucency.
  ◦ Free‐βhCG only is correlated with creatinine levels, then the FPR mainly depends on kidney function.

## 1 Introduction

For women with end‐stage renal disease, kidney transplantation is the best option for improving their quality of life. Since early studies, it is now well documented that sexual function improves and fertility is restored within the first year after transplantation, enabling successful pregnancies [1, 2, 3]. The first pregnancy following a kidney transplant was documented in 1963 [4], and since then, thousands of pregnancies have been closely monitored [5, 6]. In the early 2000s, non‐invasive screening for fetal aneuploidies (trisomy 21 [T21], 18 and 13) has become an essential part of the first trimester prenatal care [7, 8]. To calculate aneuploidy risk, screening practices mostly collect variables between 11^+0^ and 13^+6^ weeks of gestation (WG) that is, maternal age, nuchal translucency (NT) measured by ultrasounds and maternal serum concentrations of free β‐human chorionic gonadotrophin (fβ‐hCG) and pregnancy‐associated plasma protein‐A (PAPP‐A) assessed by specific immunoanalytical platforms. These concentrations are adjusted to maternal characteristics (mainly weight, ethnicity and smoking status) and to gestational age (by conversion to multiple of median or MoM). The performance generally reported for T21 screening practices was a detection rate of over 85%–90% of affected fetuses with a false positive rate between 3% and 5% [9, 10]. It has been known for a long time that the clearance from the maternal blood of the human choriogonadotropin (hCG) and its two sub‐units depends partly on renal excretion [11]. It was then expected that their serum concentration would be dependent on renal function. Thus, the first observation in 1997 of a high incidence of false‐positive mid‐term biochemical screening for T21 in a series of pregnant women who had undergone renal transplantation was not surprising [12]. This rate of false‐positives was corroborated by a few small series of second‐trimester [13] or first‐trimester [14, 15] T21 screenings. These four studies, which involved 64 posttransplant pregnancies over a period of 25 years, agreed on the elevation of fβ‐hCG MoMs and their correlation with serum creatinine. For second‐trimester screening, AFP MoMs were found to be slightly but significantly higher than those of matched controls. However, the two studies on first‐trimester screening reported discrepant results for PAPP‐A MoMs. Furthermore, due to a lack of data, it is still not possible to thoroughly evaluate the performance of T21 first‐trimester screening. This evaluation is crucial to provide a tailored prenatal care to this specific population.

The aim of the present study was to assess the performance of the first‐trimester T21 maternal serum screening as utilized in our center and to compare its features to those of previously reported in the literature. Furthermore, since prenatal cell‐free DNA screening is now implemented in many countries as a contingent (second‐tier) or as a first‐tier screening test, we will discuss its potential as an alternative in this specific setting.

## 2 Methods

This was a monocentric retrospective nested case‐control and observational study derived from the DROP cohort for the study of the first trimester prenatal screening [16]. From January 2017 to December 2023, we identified pregnant women with a history of kidney transplantation and who underwent a combined first‐trimester screening. Pregnancies with stillbirth or interruption for medical reasons were excluded. Thirty normal pregnancies matched for gestational age, age and weight were included as controls. All demographic and clinical data of cases were retrieved from hospital and laboratory records. Fβ‐hCG and PAPP‐A were assayed on a Thermo Fisher Kryptor analyzer. Creatinine was routinely assayed using Roche Cobas analyzers. Preconceptional and pregnancy reference values used in this study were those of Harel et al. [17]. Combined first‐trimester T21 risk calculation was performed using the FMF‐DE based Fastscreen software. The risk cut‐off was regulatorily fixed at 1/1000. Non‐parametrical statistical tests for quantitative and qualitative data including correlation and linear regression (cf tables and figure legends) were performed using SigmaStat software. The manuscript was drafted in accordance with the STROBE statement [18].

## 3 Results

The characteristics of cases and controls are presented in Table 1.

**TABLE 1:** Clinical and T21 screening data for controls and cases.

	Controls	Cases	*p*
Patients	30	12	—
Pregnancies	30	16	—
Age (y)^a^	32.4 (34.5–35.2)	32.0 (29.9–34.5)	0.926^b^
Weight (Kg)^a^	62.0 (56.0–79.0)	60.8	0.881^b^
Time from graft (y)^a^	—	5.1 (2.5–7.0)	—
Preconceptional creatinine (μmol/L)^a^	—	100 (79–132)^c^	—
Creatinine at first trimester (μmol/L)^a^	—	93 (83–114)^d^	—
Gestational age at screening (days)^a^	87 (85–90)	89 (87–91)	0.128^b^
NT (mm)^a^	1.35 (1.20–1.58)	1.50 (1.30–1.63)	0.404^b^
NT (MoM)^a^	0.9 (0.8–1.0)	1.05 (0.90–1.20)	0.148^b^
fβ‐hCG (ng/mL)^a^	32.2 (23.4–46.6)	74.6 (52.1–106)	**< 0.001** ^b^
fβ‐hCG (MoM)^a^	0.92 (0.67–1.41)	2.02 (1.70–2.61)	**< 0.001** ^b^
PAPPA (UI/L)^a^	3.90 (2.28–5.71)	4.26 (2.12–5.80)	0.991^b^
PAPPA (MoM)^a^	1.02 (0.64–1.29)	1.03 (0.6–1.78)	0.564^b^
T21 risk^−1^ ^a^	8168 (1933–10000)	1292 (332–7638)	**0.005** ^b^
Screened positive^e^	5 (*16.7*)	7 (*43.8*)	0.077^f^
T21 cases	0	0	—

Abbreviations: MoM, multiple of median; NT, nuchal translucency; Q, quartile.

^a^ Median (Q1‐Q3).

^b^ Mann‐Whitney U test (continuous variable). Bold values = significant.

^c^ Missing for 3 pregnancies.

^d^ Missing for 6 pregnancies.

^e^ Number (*%*), cut‐off: 1/1000.

^f^ Fisher's exact test (categorical variable).

**FIGURE 1:**
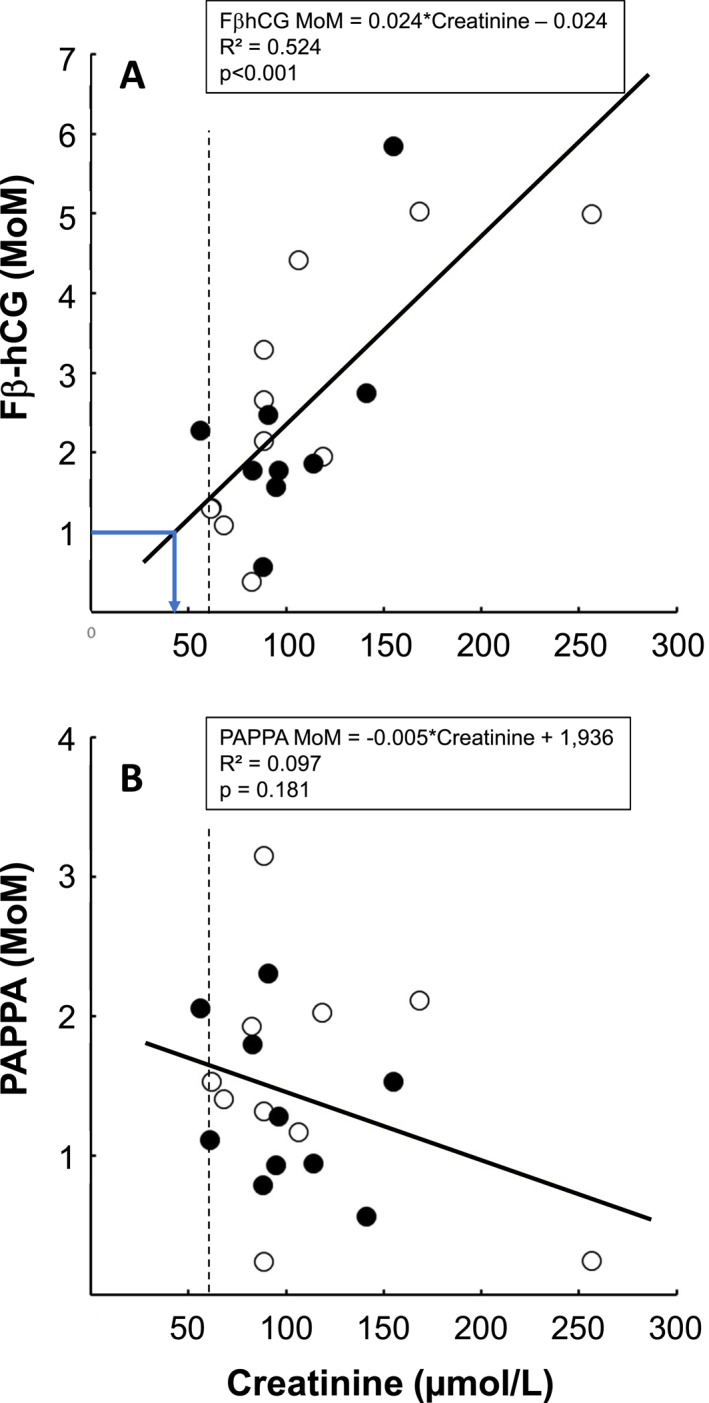
Linear Regressions of placental biomarkers (MoM) according to blood creatinine (μmol/L) in a pooled group of 20 pregnancies (black dots from this study; white dots from Grande et al.). The dashed line represents the 95^th^ percentile of creatinine levels during the first trimester of pregnancy. (A) Linear regression for fβ‐hCG; the blue line represents the expected creatinine level for 1 MoM of fβ‐hCG. (B) Linear regression for PAPP‐A.

During the study period, 12 kidney transplanted patients were screened for Down Syndrome (DS), including four who were screened for two consecutive pregnancies. The median age at the time of pregnancy was 32.0 years, which was not significantly different from that of the 30 controls. The median time from transplantation to pregnancy was 5.1 years. For grafted patients, the median values for preconceptional and first trimester (T1) serum creatinine levels were 100 and 93 μmol/L, respectively. The correlation observed between both creatinine concentrations was significant (*r* = 0.767; *p* = 0.026). No comparison with those of controls was possible. However, according to the literature, notably Harel et al. [17], both values exceeded those reported for the preconceptional period and the 10–14 gestational weeks window (where the 95^th^ percentile equals 77 and 60 μmol/L, respectively). The median values of fβ‐hCG MoM in transplanted women differed significantly from those of the control group (2.02 vs. 0.92; *p* < 0.001) whereas those of PAPP‐A and NT MoM did not. DS risk was significantly higher in transplanted women (1292 vs. 8168; *p* = 0.005) and the false‐positive rate was 2.6‐fold higher (43.8% vs. 16.7%; *p* = 0.077), though not significantly, due to the small number of cases reported. No births with DS were reported. A positive and negative Spearman correlation was found between T1 creatinine and respectively fβ‐hCG MoM and PAPP‐A MoM although not significant (correlation parameters reported in Table 2). However, a significant linear regression was observed for fβ‐hCG MoM and T1 creatinine (*r* = 0.699, *p* = 0.025 for *n* = 10). In a further step, we compared our cohort to that of Grande et al. [14] (Table 2). They retrospectively collected first trimester DS screening data of 11 transplanted pregnant women over a period of 11 years along with some of a matched‐group of 70 controls. Due to a lack of data, we could not statistically compare both control populations, but their medians of fβ‐hCG MoM and PAPP‐A MoM were very close. Since individual data of the Grande's cases were available, we recalculated medians and IQR and performed ad hoc statistical tests to compare their variables with those of our cases (Table 2). The two groups did not significantly differ in terms of age, transplantation to pregnancy time, or T1 creatinine. Their medians of NT, fβ‐hCG and PAPP‐A MoM were equivalent and their SPR at both cut‐offs (1/250 and 1/1000) were high and not significantly different. Grande et al. retrieved, as us, a positive and negative Spearman correlations of T1 creatinine with fβ‐hCG MoM and PAPP‐A MoM, respectively, but unlike us, they were significant. As there were no statistically significant differences between the two case groups in any of the tested variables, the null hypothesis (H0) that both groups derive from a single population is valid. Therefore, we were able to merge both groups into one of 27 cases for which we recalculated medians and IQR (Table 2). MoM medians for fβ‐hCG, PAPP‐A and NT were respectively 2.15, 1.28 and 1.0, depicting a very specific profile. SPR for a cut‐off of 1/250 and 1/1000 became 22.2% and 40.7%, respectively, with no T21 case reported. Then, we could pool 20 pregnancies to perform linear regression tests (Figure 1A and 1B). We found a high, positive and significant correlation between T1 creatinine and fβ‐hCG MoM (*r* = 0.724, *p* < 0.001) (Figure 1A). Of interest, the extrapolation of T1 creatinine from a normal fβ‐hCG of 1 MoM gave a result of 42.7 μmol/L, which is close to the median level for normal pregnancy at 10–14 GW that is 47 μmol/L [17]. However, we did not find any correlation between T1 creatinine and PAPP‐A MoM (*r* = 0.311, *p* = 0.181) (Figure 1B).

**TABLE 2:** Comparison and pooling of data from the present study with those from Grande et al.

	Grande et al.	This study	*p*	Pooling
General features				
Type	Retrospective cases ‐ controls	Nested cases‐controls	—	—
Period	2001–2011	2017–2023	—	2001–2023
Analytical platform	Perkin	ThermoFisher	—	—
DS screening software	LifeCycle	Fastscreen	—	—
Controls^a^				
Size	70	30	—	NA
fβ‐hCG (MoM)	0.94	0.92	—	NA
PAPPA (MoM)	1.07	1.02	—	NA
Cases				
Pregnancies (n)	11	16	—	27
Age (y)^b^	35 (6.9)	32 (4.6)	0.656^c^	32.0 (5.7)
Transplantation to pregnancy time (y)^b^	5.0 (3.0)	5.1 (4.5)	0.941^c^	5.0 (3.7)
T1 creatinine (μmol/L)^b^	88.4 (31.2)^d^	93 (31.0)^e^	0.850^c^	89.7 (32.3)^h^
fβ‐hCG (MoM)^b^	2.15 (2.72)	2.02 (0.94)	0.902^c^	2.15 (1.36)
PAPPA (MoM)^b^	1.41 (0.87)	1.03 (1.10)	0.336^c^	1.28 (1.03)
NT (MoM)^b^	0.95 (0.45)	1.05 (0.30)	0.569^c^	1.0 (0.3)
SPR (%) at 1/250	3/11 (27.3)	3/16 (18.8)	0.662^f^	6/27 (22.2)
SPR (%) at 1/1000	4/11(36.4)	7/16 (43.8)	1.000^f^	11/27 (40.7)
T21 cases	0	0	—	0
Correlation to T1 creatinine^g^				
fβ‐hCG (MoM)	0.721[10], 0.05	0.536[10], 0.20	—	Cf. Figure 1
PAPPA (MoM)	−0.812[10], 0.01	−0.370[10], 0.50	—	

Abbreviations: IQR, interquartile range; MoM, multiple of median; NA: non‐available; NT, nuchal translucency.

^a^ Comparison was not possible since some variables (age, weight, NT) or individual data were missing (biomarkers MoM).

^b^ median (IQR).

^c^ Mann‐Whitney test.

^d^ one case missing.

^e^ six cases are missing.

^f^ Fisher exact test.

^g^ Spearman correlation parameters: coefficient r_s_ [*n* = size], *p.*

^h^ *n* = 20.

## 4 Discussion

In this study, we analyzed serum creatinine levels and the results of first‐trimester DS screening in 16 pregnancies of 11 kidney‐transplanted women and highlighted several key‐points. First, the profile of screening variables was unique. We found that cases presented fβ‐hCG MoM median twice as high as that of controls (2.02 vs. 0.92, *p* < 0.001), while those of NT and PAPP‐A did not differ (Table 1). Second, SPR at the cut‐off of 1/1000 of cases was higher (43.8%) than that of controls (16.7%) but not significantly due to a lack of statistical power. In order to increase this power, we merged our data with those of Grande et al. (after statistical checking of variable equivalence). We confirmed that a group of 27 post‐transplantation pregnancies had an unusual but very specific profile: high levels for fβ‐hCG MoM but normal PAPP‐A and NT. Third, we confirmed on this larger sample a high and positive correlation of T1 creatinine with fβ‐hCG MoM but not that with PAPP‐A. The last key‐point is the insight that we provided on renal function of transplanted patients during the first trimester of pregnancy. For most of them, creatinine levels were above the 95^th^ percentile of the gestational age (Figure 1). We found a median creatinine level of 93 μmol/L, which is consistent with those of previous studies that reported values between 101 and 112 μmol/L [14, 15]. The preconceptional creatinine levels of our cases were also abnormally high (Table 1). Overall, these data confirm that the renal function remained suboptimal in the grafted patients but it does not seem to interfere with conception and early pregnancy outcomes. Interestingly, we found that both preconceptional and T1 creatinine levels were correlated (data not shown), suggesting the possibility of predicting the first trimester renal function before pregnancy.

Early studies reported on T21 screenings for pregnant women with kidney transplantation in the second trimester [12, 13]. They had already found an increased SPR at 1/250 and that fβ‐hCG MoM levels were higher in cases than in controls and positively correlated with creatinine levels. At that time, the clinical importance of those results had been pointed out as they could lead to irrelevant amniocentesis. More recently, a retrospective study explored a first‐trimester T21 screening performed using the Roche assay on a group of 27 transplanted patients [15]. As the authors did not provide individual data, it was not possible to pool them with ours and those of Grande et al. For cases, they found higher fβ‐hCG and PAPP‐A MoM but normal NT. The SPR at 1/300 was of 25.9%, which is close to our combined SPR (22.2% at 1/250). As our study showed, they reported a positive correlation of T1 creatinine with fβ‐hCG MoM but not with PAPP‐A MoM. Of interest, in a series of 55 cases of women with kidney diseases, Valentin et al. [19] showed an increased first trimester fβ‐hCG levels correlative to those of creatinine but did not notice any significant variation of PAPP‐A or NT with renal function. The consequence was a significantly increased SPR at 1/250, especially in the subgroup with confirmed renal failure (44% and 10% for cases and controls, respectively, performed on Perkin platform). At this cut‐off, the SPR of our merged population was half that of theirs (22.2%) but one's should notice that Valentin et al. reported higher medians for fβ‐hCG MoM (5.37) and creatinine (115 μmol/L) and lower for PAPP‐A (0.98) than ours. Of interest, a very recent study reported that liver‐transplanted pregnant patients display elevated fβ‐hCG with normal PAPPA during first trimester‐screening [20] and suggested that these alterations may be linked to frequent coexisting renal impairment in this specific population.

In addition to the previous study, our work has several implications. The high false‐positive rate for T21 screening was confirmed in renal‐transplanted women. Thus, in countries where amniocentesis is directly used as a second‐line invasive diagnostic test, there will be unnecessary risks and economic costs. Moreover, as Spencer et al. previously stated [13], impaired renal function falsely increases fβ‐hCG MoM and would mask the low levels normally seen in cases of trisomy 18 leading to false‐negative screenings. Both issues raise questions about using non‐invasive prenatal testing (NIPT) based on cell‐free fetal DNA (cffDNA) as a first‐line for aneuploidies screening in this specific population. NIPT is increasingly recognized as a high‐performance test [21] and it has recently been recommended by the Society for Maternal‐Fetal Medicine as the most sensitive and specific screening test for common fetal aneuploidies (trisomies 21, 18, and 13) in any patient population [22]. However, some studies conducted in different settings have revealed challenges associated with NIPT in pregnant women who have undergone a solid organ transplant [23, 24, 25]. They demonstrated three types of circulating cell‐free DNA: maternal, cffDNA and donor‐derived cell‐free DNA (dd‐cfDNA), a combination that might impair both fetal sex and aneuploidy assessment. Moreover, the use of cell‐free DNA as a biomarker is complicated by two issues: the impact of liver or renal functions on cfDNA levels is still unclear [26, 27] and measuring dd‐cfDNA remains tricky [28, 29]. Thus, the performance of NIPT in this specific setting still requires further investigation. The Society for Maternal‐Fetal Medicine identified organ transplantation as a potential source of false‐positive results and recommended genetic counseling to discuss the nuances of NIPT [22].

Second implication: since fβ‐hCG MoMs depend heavily on creatinine levels, it would be possible to adjust them in risk‐calculation software to correct the SPR [14]. However, validating this adjustment will be challenging due to the limited number of screened patients documented in the literature (only 54 patients between 2013 and 2025). A sustained international effort (i.e.., a register) is then needed. A last implication is that the profile we have identified in the cases population (high fβ‐hCG MoM but normal PAPP‐A and NT MoM) confirms the link between elevated fβ‐hCG and kidney function in non‐transplanted pregnant women that has been previously reported [30]. Thus, it would be appropriate to measure blood creatinine levels in front of this profile, to detect a potential renal failure in the first trimester of pregnancy.

A strength of our study is that we could add to the literature some first‐trimester T21 screenings in post‐transplantation pregnancies and compare them to matched‐controls. We also confirmed a high SPR on a third analytical platform (ThermoFisher besides Roche and Perkin). Limitations of our study are: its retrospective design, sample size, and the absence of Down syndrome cases that may hamper SPR refining.

In conclusion, our study confirmed the very specific profile of placental biomarkers and NT in first‐trimester post‐transplantation pregnancies as well as the impact of renal function, through fβ‐hCG levels, on the biochemical T21 screening. However, further studies are still needed to address the issues surrounding its alternatives.

## Funding

The authors have nothing to report.

## Ethics Statement

This research was derived from the DROP cohort for the study of the first trimester prenatal screening, which was approved by the research ethics committee for research of the Toulouse University Hospital in 2016 (authorization number: 08–0213). As no additional data has been collected since then, no further ethical approval is required nor is further patient consent needed for this study.

## Conflicts of Interest

SMH received conference fees from ThermoFisher.

## Data Availability

The data that support the findings of this study are available on request from the corresponding author. The data are not publicly available due to privacy or ethical restrictions.
